# Composition and Antibacterial Activity of *Aronia melanocarpa* (Michx.) Elliot, *Cornus mas* L. and *Chaenomeles superba* Lindl. Leaf Extracts

**DOI:** 10.3390/molecules25092011

**Published:** 2020-04-25

**Authors:** Magdalena Efenberger-Szmechtyk, Agnieszka Nowak, Agata Czyżowska, Alicja Z. Kucharska, Izabela Fecka

**Affiliations:** 1 Institute of Fermentation Technology and Microbiology, Lodz University of Technology, Wolczanska 171/173, 90-924 Lodz, Poland; agnieszka.nowak@p.lodz.pl (A.N.); agata.czyzowska@p.lodz.pl (A.C.); 2 Department of Fruit, Vegetable and Plant Nutraceutical Technology, Wrocław University of Environmental and Life Science, Chełmońskiego 37, 51-630 Wrocław, Poland; alicja.kucharska@upwr.edu.pl; 3 Department of Pharmacognosy and Herbal Medicines, Wrocław Medical University, Borowska 211A, 50-556 Wrocław, Poland; izabela.fecka@umed.wroc.pl

**Keywords:** polyphenols, iridoids, ellagitannins, *Aronia melanocarpa*, *Chaenomeles superba*, *Cornus mas*, antibacterial activity

## Abstract

The purpose of this study was to investigate the composition of leaf extracts from *Aronia melanocarpa*, *Chaenomeles superba*, and *Cornus mas*, and their antimicrobial activity against typical spoilage-causing and pathogenic bacteria found in meat and meat products. The highest total phenolic content (TPC) was detected in *C. superba* extract, followed by *C. mas* and *A. melanocarpa* extracts. The antioxidant capacity of the extracts was measured by DPPH and ABTS assays. The lowest IC_50_ values were found for *C. superba* extract, followed by *C. mas* and *A. melanocarpa* extracts. LC-MS and HPLC analysis revealed that *A. melanocarpa* and *C. superba* extracts contained hydroxycinnamic acid derivatives and flavonoids (mainly flavonols). Hydroxycinnamic acid derivatives were detected in the *C. mas* extract, as well as flavonols, ellagitannins, and iridoids. The antibacterial activity of the plant extracts was tested against Gram-negative bacteria (*Moraxella osloensis*, *Pseudomonas fragi*, *Acinetobacter baumanii*, *Escherichia coli*, *Enterobacter aerogenes*, *Salmonella enterica*) and Gram-positive bacteria (*Enterococcus faecium*, *Staphylococcus aureus*, *Brochothrix thermosphacta*, *Lactobacillus sakei*, *Listeria monocytogenes*) using the microculture method. The extracts acted as bacteriostatic agents, decreasing the growth rate (µ_max_) and extending the lag phase (t_lag_). *C. mas* showed most potent antibacterial activity, as confirmed by principal component analysis (PCA).

## 1. Introduction

Due to its high water activity and content of nutrients, meat is a favorable environment for the growth of microorganisms. The main microbial populations associated with the spoilage of meat and meat products belong to the genera *Bacillus*, *Pseudomonas*, *Carnobacterium*, *Enterococcus*, *Staphylococcus*, *Acinetobacter*, *Brochothrix*, *Lactococcus*, *Lactobacillus*, *Leuconostoc*, *Moraxella*, *Aeromonas*, *Acinetobacter*, *Arthrobacter*, *Flavobacterium*, and *Escherichia* [1,2,3]. The pathogenic bacteria *L. monocytogenes*, *Salmonella* sp., *Yersinia enterocolitica*, and *Staphylococcus aureus*, pathogenic strains of *E. coli* and *Pseudomonas aeruginosa*, can also be detected in meat [4].

To inhibit the growth of microorganisms and spoilage processes, manufacturers add chemical food preservatives, including nitrates. Increasing consumer awareness of the use of chemical preservatives in food is driving a demand for healthier alternatives. Researchers are considering natural methods of food preservation, including substances of animal origin (the peptides pleurocidin, defensins, and lactoferrin, as well as chitosan, lysozyme, and lipids), mushroom origin (fatty acids, polyphenols, lycopene, and polysaccharides), algae origin (fatty acids, steroids, and polyphenols), and microbial origin (the bacteriocins nisin and pediocin). However, plant substances, such as essential oils and extracts rich in bioactive compounds (polyphenols, saponins, and iridoids), have attracted the greatest attention [5,6,7]. Essential oils, due to their antioxidant and antibacterial properties, have been applied in meat products [8,9], but their organoleptic properties are a contentious issue.

Recently, polyphenolic extracts have been the subject of particular interest, due to their strong antimicrobial and antioxidant activity as well as health benefits. Polyphenols can be extracted from any part of a plant, including the fruits, buds, flowers, and leaves. However, the largest quantities are found in the leaves [10,11,12]. Therefore, leaf extracts have the potential to effectively inhibit the growth of microorganisms. So far, most studies have focused on tea leaf extracts [13], as well as some herbs and spices [14,15]. The antimicrobial activity of leaf extracts from fruit trees and shrubs has not been studied extensively. Extracts from olive leaves [16,17], walnut leaves [18], grape leaves [19], and eucalyptus leaves [20] have been investigated as potential antimicrobial agents.

Natural plant extracts have been applied in meat products to protect them from spoilage processes, extend their freshness, and inhibit the growth of pathogenic bacteria. Polyphenolic extracts have been found to prevent microbial growth, discoloration, lipid oxidation, and organoleptic changes, thereby improving meat quality. Leaf extracts have been suggested as an alternative to synthetic substances [21,22]. Cherry and blackcurrant [23], olive [24,25], mint [26], *Phyllanthus acidus* [27], and myrtle [28] leaf extracts have been used as natural preservatives in meat products.

The aim of this study was to investigate the antimicrobial activity of *Aronia melanocarpa*, *Cornus mas*, and *Chaenomeles superba* leaf extracts against typical spoilage and pathogenic bacteria found in meat and meat products. The precise compositions of the analyzed extracts were also studied, including the content of polyphenols and iridoids.

## 2. Results and Discussion

### 2.1. Composition and Antioxidant Activity of the Leaf Extracts

The highest total phenolic content (TPC) was detected in *C. superba* extract (31105.9 µg/mL), followed by *C. mas* (18676.6 µg/mL) and *A. melanocarpa* extracts (8615.6 µg/mL). The extracts also showed strong antioxidant properties. The *C. superba* extract had the lowest IC_50_ values, according to both ABTS and DPPH assays (ABTS: IC_50_ = 6.2%; DPPH: IC_50_ = 4.8%), followed by *C. mas* (ABTS: IC_50_ = 6.8%; DPPH: IC_50_ = 8.7%) and *A. melanocarpa* (ABTS: IC_50_ = 44.6%; DPPH: IC_50_ = 18.2%) extracts (Table 1).

We identified 15 phenolic compounds in the *A. melanocarpa* leaf extract (Table 2), including 4 phenolic acids and 11 flavonoids (flavonols). Among the phenolic acids we identified neochlorogenic, chlorogenic, caffeoyldeoxyhexose and *p*-coumaroylquinic acids. Unidentified hydroxycinnamic acid derivatives **1** ([M − H]^−^ = 391), **2** ([M − H]^−^ = 461) and **3** ([M − H]^−^ = 705) were also detected. The *A. melanocarpa* extract contained flavonoids from the flavonols group: quercetin derivatives (quercetin-3-*O*-rhamnoside, quercetin-pentoside-deoxydihexoside, quercetin-3-*O*-dihexoside, quercetin-3-*O*-dirhamnosylhexoside, quercetin-3-*O*-vicianoside, quercetin-3-*O*-robinobioside, quercetin-3-*O*-rutinoside, quercetin-3-*O*-glucoside), isorhamnetin derivatives (isorhamnetin-3-*O*-rutinoside, isorhamnetin-hexoside-pentoside), and kaempferol-3-*O*-rutinoside. Interestingly, we identified hydroxytyrosol (a non-flavonoid compound typically found in olive extracts [29] for the first time in *A. melanocarpa*. In the *A. melanocarpa* leaf extract, the total concentration of phenolic acids (114.66 µg/mL) was higher than that of flavonoids (93.40 µg/mL) (Table 3).

Teleszko and Wojdyło [12] identified only one additional phenolic compound, belonging to the flavan-3-ols (epicatechin), in *A. melanocarpa* leaves. Lee et al. [30], Tian et al. [31], Szopa et al. [32], and Skupień et al. [33] also found the following phenolic compounds from the group of phenolic acids: dicaffeoylquinic, 3,4-dihydroxyphenylacetic, protocatechuic, caffeic, and *p*-coumaric acids. In addition, they detected the following flavonols: apigenin-7,4-di-*O*-rhamnoside, quercetin-3-*O*-galactoside, quercetin, quercetin-deoxydihexoside-deoxyhexoside, quercetin-deoxydihexoside, quercetin rhamnosylhexoside, quercetin-arabinoglucoside kaempferol-coumaroylglucoside, kaempferol-hexoside-pentoside, isorhamnetin-rhamnosylhexoside isomers, and the non-flavonoid phenolic compound 4-(2-hydroxyethyl)phenolhexoside. Chlorogenic acid isomers were predominant in *A. melanocarpa* leaves, which is in agreement with our results. We found chlorogenic acid to be predominant, constituting 19.66% of all the compound present, followed by neochlorogenic acid at 11.35% (Table 3). Tian et al. [31] found similar percentages of chlorogenic acid isomers in *A. melanocarpa* leaves. In their study, chlorogenic acid constituted 17.75% and neochlorogenic acid 10.07% of all identified phenolic compounds. In our study, a quercetin derivative (quercetin-3-*O*-vicianoside) appeared in the highest concentration among the flavonoids and constituted 10.10% of all the presented compounds (Table 3). This is in agreement with the literature, where the highest concentrations of quercetin derivatives, such as quercetin-3-*O*-rutinoside [30], quercetin-3-*O*-arabinoglucoside, quercetin-3-*O*-rutinoside, quercetin-3-*O*-galactoside [31], and quercetin [33] were detected in *A. melanocarpa* leaves.

In *C. superba* leaf extract (Table 2), we identified 6 phenolic acids: neochlorogenic, caffeic acid dimer/caffeoyl hexoside, chlorogenic, *p*-coumaroylhexoside isomer 1, *p*-coumaroylquinic acid isomers 2 and 3, and one unidentified hydroxycinnamic acid derivative 4 ([M − H]^−^ = 431). Among the flavonoids, flavonols (dihydroquercetin-hexoside, quercetin-3-*O*-rutinoside, quercetin-3-*O*-galactoside, quercetin-3-*O*-glucoside, kaempferol-3-*O*-hexoside, kaempferol-3-*O*-rutinoside, kaempferol-hexoside-deoxyhexoside), flavanones (naringenin-7-*O*-hexoside), and flavones (luteolin-3-*O*-rutinoside, luteolin-dihexoside) were found, as well as hydroxytyrosol, a non-flavonoid phenolic compound also detected in *A. melanocarpa* extract.

To our knowledge, this is the first study of the composition of *C. superba* leaf extract. *C. superba* is a mixture of *Chaenomeles japonica* (Japanese quince) and *Chaenomeles speciose* (flowering quince). Therefore, some similarities in terms of phenolic composition may exist. *Chaenomeles japonica* leaves have been studied by Teleszko and Wojdyło [12] and Urbanaviciute et al. [34]. Similarly to our results for *C. superba*, the *C. japonica* leaves were found to contain chlorogenic acid and quercetin derivatives (galactoside, rutinoside, glucoside). In addition, *p*-coumaric acids were detected, as well as the flavan-3-ols catechin and epicatechin, and procyanidins B1, B2, B3, and C1. Ponder and Hallmann [35] and Turkiewicz et al. [36] characterized the fruits of *C. superba* and detected phenolic acids (gallic, chlorogenic, cryptochlorogenic, caffeic, *p*-coumaric acids), flavonols (quercetin-3-*O*-rutinoside, myrycetin, quercetin, luteolin), and flavan-3-ols (catechin, epicatechin, procyanidins B2, B3, and C1, and unidentified procyanidin dimers, trimers, and tetramers). However, in our study no flavan-3-ols were detected, including procyanidins, which may be due to the different extraction methods used. Turkiewicz et al. [36] and Teleszko and Wojdyło [12] used methanol/water/acetic acid/ascorbic acid as a solvent, whereas we extracted the polyphenols with water. Water is not commonly used to extract flavan-3-ols, although Dhanani et al. [37] indicate that catechin, procyanidin B2, epicatechin, epigallocatechin gallate, and epicatechin gallate can be successfully extracted with water. Therefore, these compounds may not appear in *C. superba* leaves. Moreover, many other factors, such as plant variety/cultivar, the part of the plant, and the growing season, can influence the compositions of plants and their extracts [7].

In the *C. superba* extract, the total amount of phenolic acids (644.16 µg/mL of leaves) was higher than the total amount of flavonoids (496.75 µg/mL). Chlorogenic acid was a major component (23.39% of all the presented compounds). Naringen-7-*O*-hexoside was the predominant flavonoid (17.76% of all the presented compounds) (Table 3). According to Urbanaviciute et al. [34], chlorogenic acid was also the predominant compound in *C. japonica* leaves, constituting 78.7–86.5% of the total polyphenols depending on the cultivar. In a study by Ponder and Hallmann [35], *C. superba*, and *C. japonica* pear-shape fruits had the highest contents of total polyphenols among the *C. superba* species. In *C. superba*, the concentration of phenolic acids (10.69 mg/100 g fresh weight) was higher than the concentration of flavonoids (7.64 mg/100 g of fresh weight). Chlorogenic acid (4.57/100 g of fresh weight) was the predominant compound, which is in agreement with the results of our study. However, Turkiewicz et al. [36] found procyanidin B2 to be the predominant compound in all tested cultivars of *C. superba* fruit.

*C. mas* has attracted great interest in recent years, due to its beneficial properties such as antioxidative and anti-inflammatory effects and rich diversity of bioactive compounds [38,39]. However, the leaves of *C. mas* have not as yet been the subject of extensive research, and little is known about their composition. In our study, we identified 22 compounds in *C. mas* leaf extract (Table 2). Phenolic acids (gallic acid and caftaric acid isomers 1 and 2, *p*-coumaroylhexoside isomer 2) and flavonols (quercetin-3-*O*-glucuronylpentoside, quercetin-3-*O*-rutinoside, quercetin-3-*O*-glucuronide, quercetin-3-*O*-glucoside, and kaempferol-3-*O*-glucuronide) were found. Unknown caffeic acid derivative 1 ([M − H]^−^ = 337) was also detected.

Interestingly, caftaric acid isomers and quercetin-3-*O*-glucuronylpentoside have not been identified previously in *C. mas* leaves. However, recently Martinović and Cavoski [40] identified caftaric acid in *C. mas* fruits where it was the predominant compound (12.24 mg/100 g) among all phenolic acids. Badalica-Petrescu et al. [41] and Milenković-Andjelković et al. [42] analyzed the phenolic composition of *C. mas* leaf extract and found several different compounds belonging to the phenolic acids (*p*-coumaric acid derivative, chlorogenic acid, *o*-coumaroylhexoside, an unknown caffeic acid derivative, unknown caffeic acid hexosides), as well as flavan-3-ols (catechin, epicatechin) and flavonols (quercetin-3-*O*-galactoside, quercetin-3-*O*-galactoside-7-*O*-rhamnoside, kaempferol-3-*O*-glucoside, isorhamnetin-7-*O*-rhamnoside, luteolin-3-*O*-glucoside). According to Milenković-Andjelković et al. [42], quercetin-3-*O*-glucoside was the predominant compound. In our study, the predominant compound was an unidentified caffeic acid derivative 1 ([M − H]^−^ = 337), which constituted 18.55% of all the presented compounds, followed by quercetin-3-*O*-glucuronide (13.68% of all the presented compounds).

We also identified ellagic acid and ellagitannins in the *C. mas* extract. These phenolic compounds had not been detected previously in *C. mas*. According to Czerwińska and Melzig [39], tannins such as cornusiins and camptothins have been identified only in *Cornus officinalis* fruit. Gunduz et al. [43] measured the content of tannins in *C. mas* fruit at different stages of maturation, and found them to constitute 0.16–0.45% of fresh weight. In our study, we identified seven ellagitannins (camptothin A isomers 1–4, cornusiin F 1 and 2, cornusiin A 1 and 2). The ellagitannins constituted 34.08% of all the presented compounds. Camptothin A 2 appeared in the highest concentration, constituting 26.0% of all ellagitannins and 8.85% of all the compounds found in the *C. mas* extract. This suggests that ellagitannins are a significant group of compounds in *C. mas* leaves and their presence needs to be investigated further.

Apart from polyphenols, we also detected iridoids (loganic acid isomers, secoxyloganin, and cornuside), which are monoterpenoid compounds with strong antimicrobial, antioxidant, anti-inflammatory, antitumor, and hepatoprotective activity [44]. Iridoids constituted 7.62% of all the presented compounds. Loganic acid isomer 2 was the predominant compound in this group, constituting 37.64% of all iridoids and 2.87% of all the presented compounds. To our knowledge, this is the first study to detect iridoids in *C. mas* leaves, although there have been previous reports of iridoids in *C. mas* fruit. Kucharska et al. [45] and Deng et al. [46] identified loganic acid, cornuside, sweroside, and loganin in *C. mas* fruit. Czyżowska et al. [47] also found loganic acid and cornuside in unripe *C. mas* fruit.

### 2.2. Antibacterial Activity of Leaf Extracts

**Figure 1:**
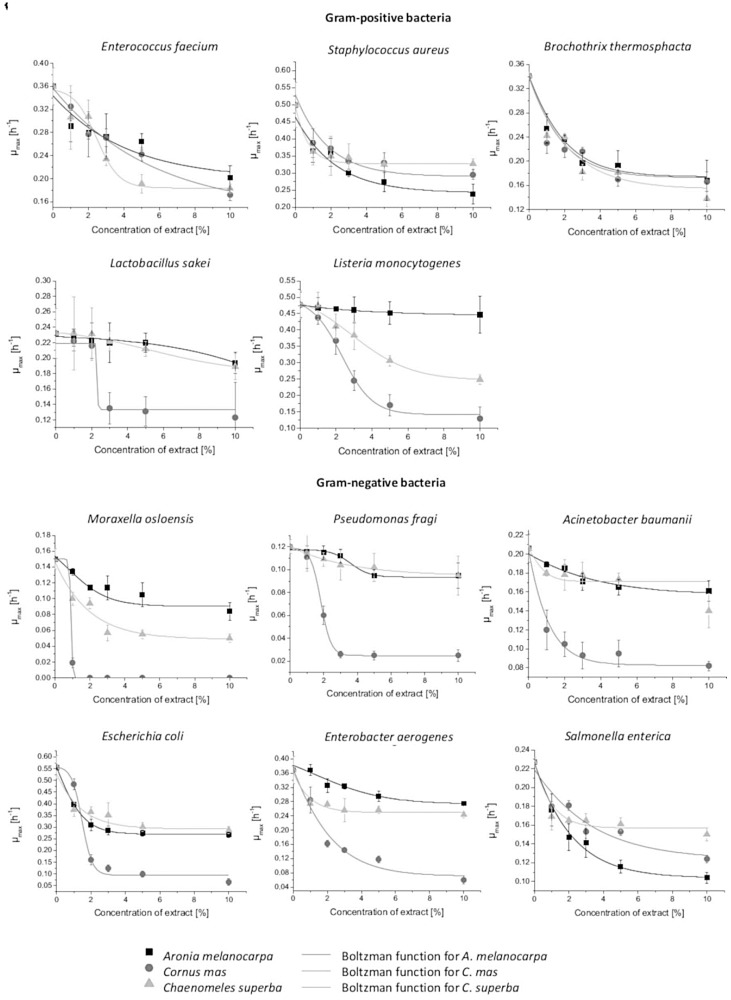
Effect of leaf extracts on bacterial growth rates (µ_max_). The results are expressed as mean ± SD.

We investigated the influence of *A. melanocarpa*, *C. mas*, and *C. superba* leaf extracts on the growth dynamics of meat spoilage bacteria. The maximum growth rate (µ_max_), time of lag phase (t_Lag_), and maximum population density (log(N_max_)) were calculated. The Gompertz function used for data analysis appropriately describes the growth of bacteria, as confirmed by the high values for the R^2^ coefficient (0.9855 to 1.000). The Boltzman function was used to evaluate the effect of several concentrations of the extracts on µ_max_. The R^2^ coefficient was in the range of 0.8902–1.0000 (Figure 1).

Our results indicate that *A. melanocarpa*, *C. superba*, and *C. mas* leaf extracts act as bacteriostatic agents. The extracts generally decreased µ_max_ and extended t_Lag_, depending on the type of extract, concentration, and bacterial strain. We observed that µ_max_ changes according to the Boltzman function and is strictly related to the extract concentration. In some cases, the extracts slightly decreased log(N_max_) and the differences were statistically significant. The greatest influence on the growth of almost all microorganisms was observed with an extract concentration of 5%. No difference was observed between using extract contents at 5% and 10% concentrations, especially regarding µ_max_ (Table 4, Figure 1, Table S1).

*C. mas* showed the highest antimicrobial activity. This suggests that antibacterial activity is not related to TPC, but rather to the composition of the extract. The *C. mas* extract contained iridoids and ellagitannins, which were not detected in the *A. melanocarpa* and *C. superba* extracts. Other studies confirm the strong antimicrobial activity of these compounds [48,49,50]. The *C. mas* extract showed the greatest inhibitory effect on *Moraxella osloensis* growth. Statistically significant changes in the growth parameters appeared already at a 1% concentration. At concentrations of 2% and higher, no bacterial growth was observed. We also noticed that the extract was more effective against Gram-negative bacteria than Gram-positive bacteria. The *C. mas* leaf extract reduced the µ_max_ of all Gram-negative bacteria at the lowest concentration (1%), except for *Pseudomonas fragi* (2%). The *C. mas* extract had a very strong influence on t_Lag_ of *P. fragi* and *Acinetobacter baumanii*, and at a concentration of 10% extended t_Lag_ for these bacteria by 23.02 h and 18.51 h, respectively. An effect was also observed on the log(N_max_) of all Gram-negative strains. Of the extracts studied, *C. mas* had the strongest inhibitory effect on the pathogenic bacteria *L. monocytogenes*, reducing its µ_max_ 3.7-fold and its log(N_max_) by 0.57 at a concentration of 10% (Table 4, Figure 1, Table S1).

Our studies are not in agreement with literature data, which suggest that Gram-positive bacteria are generally more sensitive to polyphenols than Gram-negative bacteria [14,51]. The cell walls of Gram-negative bacteria are covered by a lipophilic outer membrane, which results in less efficient permeability to hydrophilic substances. Moreover, enzymes in the periplasma space can damage molecules introduced from outside [52,53]. However, polyphenols can disintegrate the outer membrane of Gram-negative bacteria, leading to increased membrane permeability [54,55]. It should be emphasized that little is known about the antimicrobial activity of *C. mas* leaf extract. *C. mas* extract contains not only polyphenols, but also iridoids. Some interactions may occur between these compounds, explaining the stronger effect on Gram-negative bacteria in comparison to Gram-positive bacteria in our study. Moreover, the literature does not exclude the possibility of antibacterial activity against Gram-negative bacteria, which suggests that antibacterial activity may rather be related to the bacterial species or strain.

Only Milenković-Andjelković et al. [42] have discussed the antimicrobial activity of *C. mas* extract obtained from leaves, reporting that Gram-positive bacteria were more sensitive than Gram-negative bacteria. The most sensitive Gram-positive strains were *Sarcina lutea*, *Listeria monocytogenes*, and *Staphylococcus aureus*. The most sensitive Gram-negative strains were *Shigella sonnei* and *Salmonella enteritidis*. Dulger and Gonuz [56] analyzed the antibacterial activity of *C. mas* extract obtained from bark. The extract showed moderate antibacterial activity compared to other plant extracts, and inhibited the growth of *S. aureus*, *Pseudomonas aeruginosa*, *Proteus vulgaris*, and *Micrococcus luteus*. No effect was observed for the bacteria *Escherichia coli*, *Klebsiella pneumoniae*, *Bacillus cereus*, *Mycobacterium smegmatis*, or *L. monocytogenes*, nor for the yeasts *Candida albicans* and *Rhodotorula rubra*.

In our study, leaf extract from *A. melanocarpa* showed the weakest antibacterial activity. *L. monocytogenes* was most resistant bacteria to *A. melanocarpa*, which had no influence on its µ_max_ and t_Lag_ at any of studied concentrations. The most sensitive bacteria to *A. melanocarpa* were *S. aureus* and *Brochothrix thermosphacta*. Of the extracts studied, *A. melanocarpa* had the strongest influence on the pathogenic bacteria *Salmonella enterica*, with statistically significant differences in µ_max_ and t_Lag_ observed even at the lowest concentration (1%). At a concentration of 10%, the extract reduced µ_max_ 2.2-fold and extended t_Lag_ by 1.06 h. No clear relationship was found between the influence of the plant extracts and whether the bacteria belonged to the Gram-positive or Gram-negative groups (Table 4, Figure 1, Table S1).

Tian et al. [57] investigated the antibacterial activity of *A. melanocarpa* leaf extract against *E. coli*, *S. aureus*, *Bacillus cereus*, *L. monocytogenes*, and *S. enterica*. Of the tested bacteria, *E. coli* showed the greatest resistance. No growth inhibition was observed when 10 µL of the extract was used. When 20 µL of extract was used, the cell suspension was reduced by 23%. Contrary to the results of our study, the most sensitive bacteria were *L. monocytogenes* and *B. cereus*, which were inhibited by 89% and 98%, respectively, by 20 µL of extract. The antibacterial activity of *A. melanocarpa* fruit extract was also studied. The extract revealed stronger activity against *E. coli* and *L. monocytogenes* compared to the leaf extract. Cvetanović et al. [58] analyzed the antimicrobial activity of *A. melanocarpa* extract from leaves, berries, and stems against two Gram-positive bacterial strains (*S. aureus*, *Bacillus subtilis*) and four Gram-negative bacterial strains (*E. coli*, *K. pneumoniae*, *Proteus vulgaris*, *Proteus mirabilis*), as well as against two fungal species (*C. albicans* and *Aspergillus niger*). The leaf extract showed the most potent activity against *P. mirabilis* (MIC = 19.53 µg/mL). The most resistant strains were *K. pneumoniae*, *E. coli* and *A. niger* (MIC = 312.5 µg/mL). Rauha et al. [59] studied the antimicrobial activity of *A. melanocarpa* berries. The *A. melanocarpa* extract inhibited the growth of *M. luteus* at a concentration of 1 mg/mL, and showed slight antimicrobial activity against *S. aureus*, *B. subtilis*, and *E. coli*, but was inactive against *Staphylococcus epidermidis* and fungi (*A. niger* and *C. albicans*).

In our study, *C. superba* extract showed slightly stronger antibacterial activity than the *A. melanocarpa* extract. The *C. superba* extract had the greatest effect on the t_Lag_ of *Lactobacillus sakei* and *P. fragi*. The t_Lag_ of *L. sakei* increased by 9.21 h and that of *P. fragi* by 9.68 h when *C. superba* was applied at a concentration of 10%. However, no effect was observed on the log(N_max_) of any of the studied bacteria (Table 4, Figure 1, Table S1). To the best knowledge of the authors, there is no data in the previous literature regarding the antimicrobial activity of C. *superba* extract obtained from any parts of the plant. Previous studies have demonstrated the antibacterial activity of *C. japonica* extracts. Leaf extracts were found to be most active against Gram-positive *S. aureus*, but also showed activity against *E. coli*, *P. aeruginosa*, and *C. albicans* [60]. More research is needed into the composition and antimicrobial activity of *C. superba*. However, *C. superba* extract shows great biological potential, due to its high content and diverse range of phenolic compounds, as well as strong antioxidant activity.

**Figure 2:**
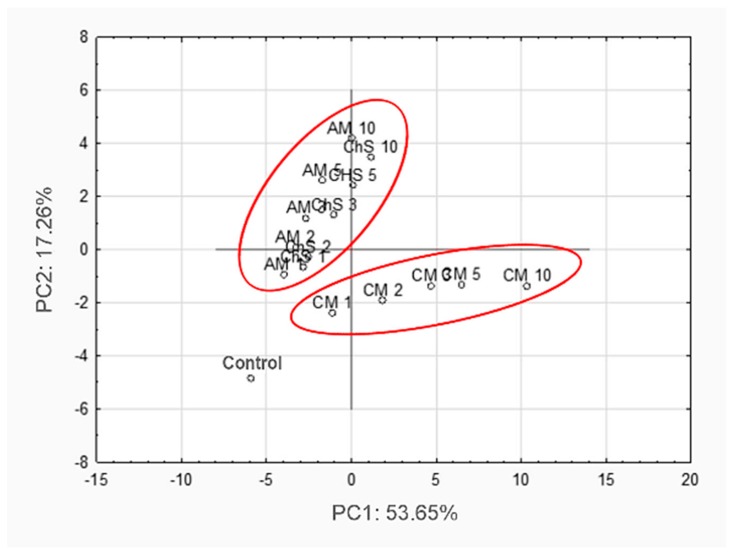
Principal component analysis (PCA) presenting the effect of leaf extracts on the growth of meat spoilage and pathogenic bacteria. (AM-*Aronia melanocarpa*, CM–*Cornus mas*, ChS–*Chaenomeles superba*; 1,2,3,5,10–concentrations of extract [%]).

Figure 2 presents a principal component analysis (PCA) of the growth parameters of all the investigated bacterial strains. PC1 and PC2 explain 70.91% of the total variance. All 34 variables had high significance for the separation of samples, with variable strengths of 0.838–0.996. Three main groups can be distinguished. All the samples with leaf extracts are separated from the control, indicating an inhibitory effect on bacterial growth. Samples with *A. melanocarpa* and *C. superba* belong to one cluster, suggesting similar antimicrobial activity. The samples with *C. mas* extracts are clearly separated from the others, confirming that *C. mas* extract had the strongest antibacterial activity.

The antibacterial activity of the extract is the result of the activity of all compounds and the interactions between them. Literature data show that flavonoids are the compounds of strong antibacterial properties. Their biological activity is well investigated. Flavonols most commonly identified in plants (quercetin, kaempferol, myricetin and its derivatives) as well as flavanones (naringenin found in citrus fruits), flavones (luteolin ), chalcones and flavan-3-ols (catechins) were reported to inhibit bacteria growth [61]. In *A. melanocarpa*, *C. mas* and *C. superba* extracts we detected high amounts of quercetin and kaempferol derivatives. In *C. superba* extract naringenin and luteolin derivatives were also identified. However, no catechins of well documented antibacterial properties were found. Phenolic acids are poorly investigated in terms of their biological activity. The antibacterial activity of chlorogenic acid—most frequently found in plants—was demonstrated [62]. Studies show that ellagitannins [63] and iridoids [64,65] which we found in *C. mas* extract also reveal antibacterial properties, but they have not been widely examined.

The cytotoxicity of plant extracts used in food products seems to be an important aspect. Depending on the concentration, the extract can be toxic, non-toxic or can show protective properties to eukaryotic cells. In addition, the extracts can be toxic to cancer cells and therefore can reveal anticancer properties. However, studies showed that the activity of extracts can be selective, inhibiting proliferation of cancer cells and not affecting normal cells [66,67]. It should be emphasized that studies focus mainly on investigating cytotoxic activity of plant extracts against cancer cells showing their health benefits. Investigating cytotoxic effect against normal cells is often omitted, especially when the edible plants are tested, and the concentrations used are low.

In summary, our studies show that *A. melanocarpa*, *C. superba*, and *C. mas* leaves constitute a rich source of bioactive compounds, mainly polyphenols from the phenolic acids and flavonoids groups. The *C. mas* extract was also found to contain iridoids. This is the first time these monoterpenoid compounds have been identified in *C. mas* leaves. Also for the first time, ellagitannins were detected in the *C. mas* leaves. The highest levels of polyphenolic compounds (TPC) were detected in *C. superba* leaf extract, which also showed the strongest antioxidant activity. We demonstrated the effect of *A. melanocarpa*, *C. superba*, and *C. mas* leaf extracts on the growth of spoilage and pathogenic bacteria found in meat and meat products. The extracts generally acted as bacteriostatic agents, influencing the bacterial growth parameters mainly by decreasing the growth rate (µ_max_) and extending the lag phase (t_Lag_). The strongest antibacterial activity was shown by *C. mas* leaf extract, followed by *C. superba* and *A. melanocarpa*. The *C. mas* leaf extract was more effective against Gram-negative bacteria than against Gram-positive bacteria. In terms of *A. melanocarpa* and *C. superba* extract, no clear relationship in Gram-staining was observed. It can be concluded that, due to the high content of bioactive compounds, strong antioxidant capacity, and antibacterial properties, *C. mas*, *C. superba*, and *A. melanocarpa* leaf extracts have great potential for use as natural preservatives in meat products.

## 3. Materials and Methods

### 3.1. Plant Material

Plant material was collected in the region of Lodz (central Poland) on 15 July 2016. Leaves from *Aronia melanocarpa* (Michx.), Elliot (black chokeberry), and *Chaenomeles superba* Lindl. were collected in the village of Tymianka, and *Cornus mas* L. (*C. mas*) leaves from the village of Zadzim. Only fully developed, healthy leaves were selected. The plant material was transported to the laboratory, washed, and stored at −20 °C prior to extract preparation.

### 3.2. Bacterial Strains

The research material comprised typical spoilage and pathogenic bacterial strains found in meat and meat products (Table 5). The microorganisms were obtained from three culture collections, the American Type Culture Collection (ATCC, Manassas, VA, USA), the Polish Culture of Microorganisms (PCM, Wrocław, Poland), and the Collection of Industrial Microorganisms at the Institute of Fermentation Technology and Microbiology (ŁOCK, Łódź, Poland). Other strains were isolated from water, chicken meat, and meat packed in a modified atmosphere. These strains were genetically identified by 16S rRNA gene sequencing, and the sequences were deposited in the GenBank Database under appropriate accession numbers.

### 3.3. Extraction of Polyphenols and Iridoids from Leaves

To extract polyphenols and iridoids, the leaves were crushed and approx. 200 g of each material was shaken for 1 h at 4 °C with 1000 mL of distilled water, then homogenized for 1 min at 20 °C. The samples were filtrated and centrifuged for 10 min at 10,000× *g* (5804R Centrifuge, Eppendorf, Hamburg, Germany). The supernatant was collected. The extracts were sterilized using microwaves (Microjet, EmbioTechnology, Oensingen, Switzerland), frozen at −20 °C and then stored prior to further analysis.

### 3.4. Total Phenolic Content

The total phenolic content (TPC) of the leaf extracts was determined using the Folin-Ciocalteu colorimetric method [68]. The reaction mixture contained the following components: 100 µL of polyphenolic extract; 200 µL of Folin-Ciocalteu reagent; 1 mL of 20% Na_2_CO_3_ solution, and 2 mL of distilled water. The blank sample contained 100 µL of distilled water instead of leaf extract. The solutions were mixed and kept for 1 h in a dark place at room temperature. The absorbance was measured at 765 nm against the blank sample using a Cecil CE2041 spectrophotometer (Cecil Instruments Limited, Cambridge, UK). The TPC was quantified according to a calibration curve prepared for gallic acid and expressed as µg_GAE_/mL.

### 3.5. Antioxidant Activity

#### 3.5.1. DPPH

Antioxidant activity was determined using the DPPH free radical scavenging assay, according to the methods described by Meda et al. [69] and Al et al. [70], with some modifications. The stock solution was prepared by dissolving 2.4 mg of 2,2-diphenyl-1-picrylhydrazyl (DPPH) (Sigma-Aldrich, St Louis, MO, USA) with 10 mL of 80% ethanol. To 1.95 mL of the DPPH free radical solution was added 50 µL of plant extract. The samples were left for 15 min in a dark place at room temperature. The absorbance was measured at 515 nm against 80% ethanol, using a Cecil CE2041 spectrophotometer (Cecil Instruments Limited, Cambridge, UK). The free radical scavenging activity determined by DPPH was expressed as the IC_50_ value (the concentration of extract required to inhibit 50% of the initial DPPH free radical).

#### 3.5.2. ABTS

The ABTS radical scavenging assay was performed as described by Re et al. [71]. ABTS (2,2′-azino-bis(3-ethylbenzothiazoline-6-sulphonic acid) (Sigma-Aldrich) was dissolved in water to a 7 mmol concentration. The ABTS radical cation (ABTS+) was obtained by reacting ABTS stock solution with 2.45 mmol K_2_S_2_O_8_ (final concentration), then kept in a dark place at room temperature for 12–16 h before use. The ABTS+ solution was next diluted to an absorbance of 0.700 at 734 nm. The reaction mixtures contained 3 mL of diluted ABTS+ solution and 30µL of plant extract. The samples were mixed and the absorbance measured after 10 min at 734 nm against distilled water. All analyses were performed in triplicate using a Cecil CE2041 spectrophotometer (Cecil Instruments Limited). The free radical scavenging activity determined by ABTS was expressed as the IC_50_ value (the concentration of extract required to inhibit 50% of the initial ABTS free radical).

### 3.6. Identification of Polyphenols and Iridoids

#### 3.6.1. Identification of Phenolic Acids and Flavonoids with LC-MSn

Polyphenolic compounds were identified according to a previously published procedure [23]. The plant extracts were filtered through 0.45µm membrane filters prior to analysis. The HPLC was coupled on-line to an MS LTQ Velos mass spectrometer (ThermoScientific, Waltham, MA, USA). Chromatographic separation was achieved with a Hypersil Gold 150 × 2.1 column, particle size 1.9 µm (Thermo Scientific) operating at 45 °C. Detection wavelengths were set to 280, 320, and 360 nm. The mobile phase was a mixture of 0.01% formic acid (solvent A) and 95% acetonitrile (solvent B). The injection volume of the sample was 10 µL and the flow rate was 220 µL/min. The overall separation time was 55 min. The gradient elution applied was as follows: 8 min, 96–85% (A); 22 min, 85–70% (A); 10 min, 70–60% (A); 4 min, 60–50% (A); 3 min, 50% (A); 2 min, 50–96% (A); 6 min, 96%. The column was then washed and re-equilibrated.

Electrospray ionization mass spectrometry was performed using an LTQ Velos mass spectrometer (ThermoScientific) equipped with an ESI interface and controlled by Excalibur software. The analysis was performed in the negative mode across a range of 120–1000 *m*/*z*. The I spray voltage was 4 kV. The sheath gas flow rate was 25 and the aux gas flow rate 10. The desolvation temperature was 280 °C. The source temperature was 350 °C. Polyphenols were identified by comparison with the retention times and mass spectra of standards.

#### 3.6.2. Identification of Ellagitannins and Iridoids by UPLC-qTOF-MS/MS

To identify ellagitannins and iridoids, the UPLC-qTOF-MS/MS method was applied, as described previously by Kucharska et al. [72]. An Acquity ultra-performance liquid chromatography (UPLC) system was used, coupled to a quadrupole-time of flight (Q-TOF) MS instrument (Waters Corp., Milford, MA, USA) with an electrospray ionization (ESI) source. Separation was achieved on an Acquity BEH C18 column (100 mm × 2.1 mm i.d., 1.7 µm; Waters). The mobile phase was a mixture of 0.1% aq. formic acid *v*/*v* (A) and acetonitrile (B). The gradient program was as follows: initial conditions, 1% B in A; 12 min, 25% B in A; 12.5 min, 100% B; 13.5 min, 1% B in A. The flow rate was 0.45 mL/min and the injection volume 5 µL. The column was operated at 30 °C. UV-Vis absorption spectra were recorded on-line during UPLC analysis. Spectral measurements were made in the wavelength range of 200–600 nm, in steps of 2 nm. The major operating parameters for the Q-TOF MS were set as follows: capillary voltage, 2.0 kV; cone voltage, 40 V; cone gas flow, 11 L/h; collision energy, 28–30 eV; source temperature, 100 °C; desolvation temperature, 250 °C; collision gas, argon; desolvation gas (nitrogen) flow rate, 600 L/h; data acquisition range, *m*/*z* 100–2500 Da. The compounds were explored in the negative mode before and after fragmentation. The runs were monitored at the following wavelengths: iridoids at 245 nm; ellagic acid at 254 nm, ellagitanins at 280 nm.

### 3.7. Quantification of Polyphenols and Iridoids

#### 3.7.1. Quantification of Phenolic Acids and Flavonoids Using HPLC-PDA

Polyphenolic compounds were quantified according to a previously published procedure [23]. Prior to analysis, the samples were filtered using 0.45 µm membrane filters. HPLC-PDA analyses were performed using a Finnigan Surveyor equipped with an autosampler and a diode array detector (Finnigan Surveyor PDA Plus Detector, Thermo Scientific), controlled with ChromQuest 5.0 chromatography software (Thermo Fisher Scientific Inc.). Separation was achieved using a Spherisorb ODS2 column (250 × 4.6 mm × 5 µm packing) (Waters), protected with a guard column of the same material. The volume of the injected sample was 50 µL, the flow rate was 0.8 mL/min, and the separation time was 60 min. The mobile phase consisted of 5% formic acid (solvent A) and 95% acetonitrile (solvent B). The samples were eluted with the following gradient: 2 min, 97% (A); 13 min, 97–85% (A); 9 min, 85–82% (A); 31 min, 82–75% (A); 5 min, 75–70% (A). Polyphenols were quantified according to calibration curves established for gallic acid (280 nm), caffeic acid (320 nm), and quercetin-glucoside (360 nm). The concentration of polyphenols was expressed as µg/g of leaves.

#### 3.7.2. Quantification of Ellagitannins and Iridoids Using HPLC-PDA

To quantify the ellagitannins and iridoids, the HPLC-PDA method used, as described previously by Kucharska et al. [72]. The analysis was performed using a Dionex (Germering, Germany) system, equipped with an Ultimate 3000 diode array detector, an LPG-3400A quaternary pump, an EWPS-3000SI autosampler, and a TCC-3000SD thermostated column compartment. A C_5_-C_18_ Cadenza Imtakt column (75 × 4.6 mm, 5 µm) was used. The mobile phase was composed of solvent A (4.5% aq. formic acid, *v*/*v*) and solvent B (100% acetonitrile). The elution system was as follows: 0–1 min 5% B in C, 20 min 25% B in A, 21 min 100% B, 26 min 100% B, 27 min 5% B in A. The flow rate of the mobile phase was 1.0 mL/min and the injection volume was 20 µL. The column was operated at 30 °C. Iridoids were detected at 245 nm, ellagic acid at 254 nm, ellagitanins at 280 nm. Isomers of loganic acid were expressed as loganic acid. Isomers of secoxyloganin and cornuside were expressed as loganin. Isomers of ellagic acid were expressed as the appropriate standards. Isomers of ellagitanins were expressed as gallic acid. All results were expressed as µg/mL.

### 3.8. Antibacterial Activity

The effect of the plant extracts on the growth of the bacteria was investigated using the microculture method (microliter plate assay). The bacterial strains were cultured for 24 h at 30 °C in a Tryptic Soy Broth (TSB) medium. The extracts were added to the media at working concentrations of 1, 2, 3, 5, and 10%. The media were inoculated with bacterial strains. The control samples consisted of media containing only polyphenolic extracts and bacterial cultures without extracts. The wells of 96 microliter plates were filled with 200 µL samples and incubated at 30 °C until stationary phase was achieved. Bacterial growth was determined by measuring absorbance at 540 nm using a microliter plate reader (Biogenet, Józefów, Poland). Calibration curves were prepared for all bacterial strains and the OD_540_ values were converted into CFU/mL.

### 3.9. Mathematical Models

Sigmoidal (*S*-shaped) curves produced by Gompertz and Boltzman functions are commonly used in biological studies to describe growth patterns. The cell numbers were fitted to the Gompertz equation using a Microsoft Excel add-in, DMFit 2.1 (Institute of Food Research, Norwich, UK): L(t) = A + C exp{−exp[−B (t − M)]}. The following growth parameters were calculated: maximum specific growth rate µ_max_ = BC/e; lag time t_Lag_ = M − (1/B); maximum population density log_(Nmax)_ = A + C.

Using the Origin 6.1. software (OriginLab, Northampton, MA, USA), a Boltzmann function producing a sigmoidal curve was fitted to describe the effect of different concentrations of plant extracts on µ_max._ The function was expressed by the following equation: y = A2 + (A1 − A2)/(1 + exp((x − x0)/*d*x)), where A1 = initial value, A2 = final value, x0 = center, *d*x = time constant.

### 3.10. Statistical Analysis

Mean values and standard deviations (SD) were calculated using Microsoft Excel 2013 (Microsoft, Waszyngton, WA, USA). Analysis of variance (one-way ANOVA) was performed using R 3.4.0 software (R Core Team, Vienna, Austria). Tukey’s Honestly Significant Differences (HSD) test was used to determine differences between variables (*p* < 0.05).

### 3.11. Chemometric Analysis

Principal component analysis (PCA) was performed using the Statistica 13 software (StatSoft, Poland, Kraków) to separate the leaf extracts, based on their inhibitory effects on the bacterial growth parameters.

## Figures and Tables

**Table 1:** Total phenolic content (TPC) and antioxidant activity in leaf extracts. The results are expressed as mean ± SD.

Extract	TPC [µg/mL]	Antioxidant Activity IC_50_ [%]	
		DPPH	ABTS
*Aronia melanocarpa*	8615.6 ± 229.8 ^c^	18.2 ± 0.50 ^a^	44.6 ± 1.23 ^a^
*Chaenomeles superba*	31105.9 ± 860.9 ^a^	4.8 ± 0.71 ^c^	6.2 ± 0.13 ^c^
*Cornus mas*	18676.6 ± 384.5 ^b^	8.7 ± 0.86 ^b^	6.8 ± 0.18 ^b^

^a,b,c^—statistically significant differences (*p* < 0.05).

**Table 2:** Identification of polyphenols and iridoids in leaf extracts.

R_t_ [min]	[M − H]^−^	MS^2^	Compound	Extract
Phenolic Acids *				
3.19	337	179	Caffeic acid derivative 1	CM
3.25	309	179;129;161	Caffeoyl-deoxyhexose	AM
3.74	169	-	Gallic acid	CM
4.42	311	149; 179	Caftaric acid isomer 1	CM
6.01	391	183; 207; 211; 167; 323	Hydroxycinnamic acid derivative 1	AM
6.69	353	179; 191	Neochlorogenic acid	AM, Ch
7.27	461	163; 177; 207; 297; 315	Hydroxycinnamic acid derivative 2	AM
7.59	705	513	Hydroxycinnamic acid derivative 3	AM
7.90	341	179; 161	Caffeic acid dimer/caffeoyl hexoside	Ch
7.95	311	149; 179	Caftaric acid isomer 2	CM
8.17	337	163	*p*-Coumaroylquinic acid isomer 1	AM
9.24	353	191, 171	Chlorogenic acid	AM, Ch
9.71	325	145; 163; 187	*p*-Coumaroylhexoside isomer 1	Ch
10.93	325	193	*p*-Coumaroylhexoside isomer 2	CM
11.25	337	191; 163	*p*-Coumaroylquinic acid isomer 2	Ch
11.88	431	341; 205; 367	Hydroxycinnamic acid derivative 4	Ch
12.46	337	191, 163	*p*-Coumaroylquinic acid isomer 3	Ch
Flavonols *				
8.76	465	303; 285	Dihydroquercetin hexoside	Ch
10.07	447	269; 401	Quercetin-3-*O*-rhamnoside	AM
13,27	741	300/301; 489; 577; 409	Quercetin-pentoside-deoxydihexoside	AM
13.37	625	300/301	Quercetin-3-*O*-dihexoside	AM
13.82	755	300/301	Quercetin-3-*O*-dirhamnosylhexoside	AM
14.42	609	301	Quercetin-3-*O*-glucuronylpentoside	CM
14.72	595	301	Quercetin-3-*O*-vicianoside	AM
14.99	593	447; 285	Kaempferol-hexoside-deoxyhexoside	Ch
15.81	609	301	Quercetin-3-*O*-robinobioside	AM
16.10	609	301	Quercetin-3-*O*-rutinoside	AM, Ch, CM
16.16	477	301	Quercetin-3-*O*-glucuronide	CM
16.42	463	301	Quercetin-3-*O*-galactoside	Ch
16.80	463	301	Quercetin-3-*O*-glucoside	AM, Ch, CM
17.58	609	315	Isorhamnetin-hexoside-pentoside	AM
18.56	593	285	Kaempferol-3-*O*-rutinoside	AM. Ch
19.19	461	285	Kaempferol-3-*O*-glucuronide	CM
19.33	623	300; 315	Isorhamnetin-3-*O*-rutinoside	AM
19.31	447	285; 327	Kaempferol-3-*O*-hexoside	Ch
Flavones *				
13.63	609	285; 447;	Luteolin-dihexoside	Ch
15.76	593	447; 431; 285	Luteolin-3-*O*-rutinoside	Ch
Flavanones *				
18.77	433	271	Naringenin-7-*O*-glucoside	Ch
Ellagitannins **				
1.325	708^−2^	633; 301; 169	Camptothin A isomer 1	CM
1.675	708^−2^	633; 301; 169	Camptothin A isomer 2	CM
1.925	708^−2^	633; 301; 169	Camptothin A isomer 3	CM
2.233	1100^−2^	633; 301; 169	Cornusin F isomer 1	CM
2.45	783^−2^	301; 169	Cornusiin A isomer 1	CM
2.683	1100^−2^	633; 301; 169	Cornusin F isomer 2	CM
2.817	708^−2^	633; 301; 169	Camptothin A isomer 4	CM
3.200	783^−2^	301; 169	Cornusiin A isomer 2	
Ellagic Acid **				
11.85	301	-	Ellagic acid	CM
Substituted Phenols *				
3.79	315	153	Hydroxytyrosol	AM, Ch
Iridoids **				
5.017	375	213	Loganic acid isomer 1	CM
6.867	375	213	Loganic acid isomer 2	CM
7.708	375	213	Loganic acid isomer 3	CM
10.533	403	223	Secoxyloganin	CM
16.625	541	169	Cornuside	CM
Unidentified Compounds *				
8.94	451	405	Unidentified 1	AM
12.47	433	387	Unidentified 2	AM
13,31	611	431; 251	Unidentified 3	AM
14.04	649	605	Unidentified 4	Ch
17.66	503	293; 457	Unidentified 5	AM

* Compounds identified with LC-MSn method according to 3.6.1. ** Compounds identified with UPLC-qTOF-MS/MS method according to 3.6.2. AM–*Aronia melanocarpa*. Ch–*Chaenomeles superba*. CM–*Cornus mas*.

**Table 3:** Quantification of polyphenols and iridoids in leaf extracts (µg/mL).

Compound	*Aronia melanocarpa*	*Chaenomeles superba*	*Cornus mas*
Phenolic Acids *			
Caffeic acid derivative 1	-	-	82.52 ± 6.60
Caffeoyl-deoxyhexose	3.65 ± 0.18	-	-
Gallic acid	-	-	1.61 ± 0.08
Caftaric acid isomer 1	-	-	29.28 ± 1.46
Hydroxycinnamic acid derivative 1	1.16 ± 0.06	-	-
Neochlorogenic acid	26.88 ± 1.88	97.23 ± 5.83	-
Hydroxycinnamic acid derivative 2	11.44 ± 0.57	-	-
Hydroxycinnamic acid derivative 3	13.41 ± 0.67	-	-
Caffeic acid dimer/caffeoyl hexoside	-	68.04 ± 5.44	-
Caftaric acid isomer 2	-	-	27.54 ± 1.65
*p*-Coumaroylquinic acid isomer 1	11.59 ± 0.81	-	-
Chlorogenic acid	46.53 ± 2.33	299.23 ± 14.96	-
*p*-Coumaroylhexoside isomer 1	-	16.66 ± 0.83	-
*p*-Coumaroylhexoside isomer 2	-	-	4.15 ± 0.21
*p*-Coumaroylquinic acid isomer 2	-	143.62 ± 7.18	-
Hydroxycinnamic acid derivative 4	-	13.72 ± 0.69	-
*p*-Coumaroylquinic acid isomer 3	-	5.66 ± 0.40	-
**Total**	**114.66 ± 6.50**	**644.16 ± 35.33**	**145.1 ± 10.01**
Flavonols *			
Dihydroquercetin-hexoside	-	84.50 ± 4.23	-
Quercetin-3-*O*-rhamnoside	5.42 ± 0.27	-	-
Quercetin-pentoside-deoxydihexoside	2.00 ± 0.12	-	-
Quercetin-3-*O*-dihexoside	10.63 ± 0.74	-	-
Quercetin-3-*O*-dirhamnosylhexoside	17.36 ± 1.22	-	-
Quercetin-3-*O*-glucuronylpentoside	-	-	19.47 ± 1.36
Quercetin-3-*O*-vicianoside	23.91 ± 1.20	-	-
Kaempferol-hexoside-deoxyhexoside	-	8.25 ± 0.41	-
Quercetin-3-*O*-robinobioside	6.02 ± 0.36	-	-
Quercetin-3-*O*-rutinoside	15.28 ± 1.07	29.02 ± 1.74	7.68 ± 0.38
Quercetin-3-*O*-glucuronide	-	-	60.88 ± 4.26
Quercetin-3-*O*-galactoside	-	96.78 ± 4.84	-
Quercetin-3-*O*-glucoside	3.12 ± 0.16		5.67 ± 0.28
Isorhamnetin-hexoside-pentoside	3.37 ± 0.17	-	-
Kaempferol-3-*O*-rutinoside	2.92 ± 0.15	15.23 ± 0.76	-
Kaempferol-3-*O*-glucuronide	-	-	17.96 ± 0.90
Isorhamnetin-3-*O*-rutinoside	3.37 ± 0.24	-	-
Kaempferol-3-*O*-hexoside	-	18.39 ± 1.29	-
**Total**	**93.40 ± 5.66**	**252.17 ± 13.27**	**111.66 ± 7.19**
Flavones *			
Luteolin-dihexoside	-	nd	-
Luteolin-3-*O*-rutinoside	-	17.28 ± 0.86	-
**Total**	-	**17.28 ± 0.86**	-
Flavanones *			
Naringenin-7-*O*-hexoside	-	227.30 ± 11.37	-
**Total**	**-**	**227.30 ± 11.37**	-
Ellagitannins **			
Camptothin A isomer 1	-	-	13.87 ± 1.54
Camptothin A isomer 2	-	-	39.38 ± 1.45
Camptothin A isomer 3	-	-	12.88 ± 0.10
Cornusin F isomer 1	-	-	22.36 ± 1.17
Cornusiin A isomer 1	-	-	19.64 ± 0.25
Cornusin F isomer 2	-	-	14.47 ± 1.02
Camptothin A isomer 4	-	-	14.01 ± 1.47
Cornusiin A isomer 2			15.02 ± 1.77
**Total**	**-**	**-**	**151.63 ± 8.77**
Ellagic Acid **			
Ellagic acid	-	-	2.56 ± 0.27
**Total**	**-**	**-**	**2.56 ± 0.27**
Substituted Phenols *			
Hydroxytyrosol	7.91 ± 0.40	75.00 ± 5.25	-
**Total**	**7.92 ± 0.40**	**75.00 ± 5.25**	-
Iridoids **			
Loganic acid isomer 1	-	-	8.52 ± 0.58
Loganic acid isomer 2	-	-	12.76 ± 0.12
Loganic acid isomer 3	-	-	2.96 ± 0.23
Secoxyloganin	-	-	6.21 ± 0.54
Cornuside	-	-	3.45 ± 0.38
**Total**	**-**	**-**	**33.9 ± 1.85**
Unidentified Compounds *			
Unidentified 1	4.02 ± 0.20	-	-
Unidentified 2	6.67 ± 0.33	-	-
Unidentified 3	6.67 ± 0.33	-	-
Unidentified 4	-	63.49 ± 3.17	-
Unidentified 5	3.29 ± 0.16	-	-
**Total**	**20.65 ± 1.03**	**63.49 ± 3.17**	**-**

The results are expressed as mean ± SD. * compounds quantified with HPLC. PDA according to 3.7.1. ** compounds quantified with HPLC-PDA according to 3.7.2. nd–not detected with HPLC.

**Table 4:** Effect of leaf extracts on the lag time (t_Lag_) and maximum population density (log_(Nmax)_) of bacteria.

		*Aronia melanocarpa*						*Chaenomeles superba*						*Cornus mas*					
Bacterial Strain	Extract Concentration [%]	t_Lag_ [h]			log_(Nmax)_			t_Lag_ [h]			log _(Nmax)_			t_Lag_ [h]			log_(Nmax)_		
**Gram-Positive Bacteria**																			
*Enterococcus faecium*	0	6.12	±	0.13 ^b^.^c^	8.85	±	0.03 ^a^	6.12	±	0.13 ^a^	8.85	±	0.03 ^a^	6.12	±	0.13 ^b^	8.85	±	0.03 ^a^
	1	6.03	±	0.02 ^b^.^c A^	8.85	±	0.05 ^a A^	6.17	±	0.13 ^a A^	8.85	±	0.04 ^a A^	6.19	±	0.09 ^a^.^b A^	8.86	±	0.03 ^a A^
	2	6.20	±	0.10 ^a^.^b A^	8.83	±	0.05 ^a A^	6.01	±	0.04 ^a A^	8.84	±	0.04 ^a A^	6.17	±	0.04 ^a^.^b A^	8.83	±	0.04 ^a A^
	3	5.98	±	0.03 ^c A^	8.80	±	0.05 ^a A^	6.13	±	0.13 ^a A^	8.86	±	0.05 ^a A^	6.17	±	0.10 ^a^.^b A^	8.83	±	0.07 ^a A^
	5	6.04	±	0.08 ^b^.^c B^	8.84	±	0.03 ^a A^	6.06	±	0.07 ^a B^	8.87	±	0.02 ^a A^	6.05	±	0.04 ^b B^	8.84	±	0.012 ^a A^
	10	6.32	±	0.06 ^a A^	8.81	±	0.04 ^a A^	6.02	±	0.03 ^a B^	8.86	±	0.02 ^a A^	6.36	±	0.01 ^a A^	8.86	±	0.02 ^a A^
*Staphylococcus aureus*	0	10.85	±	0.32 ^b^	9.47	±	0.04 ^a^	10.85	±	0.32 ^b^	9.47	±	0.04 ^a^	10.85	±	0.33 ^b^	9.47	±	0.04 ^a^
	1	10.85	±	0.08 ^b B^	9.44	±	0.07 ^a A^	10.75	±	0.32 ^b B^	9.43	±	0.02 ^a A^	12.07	±	0.89 ^ab A^	9.45	±	0.04 ^ab A^
	2	11.13	±	0.35 ^b AB^	9.45	±	0.08 ^a A^	10.62	±	0.33 ^b B^	9.41	±	0.04 ^a A^	11.97	±	0.43 ^ab A^	9.41	±	0.02 ^ab A^
	3	10.95	±	0.30 ^b A^	9.44	±	0.03 ^a A^	11.78	±	0.97 ^b A^	9.40	±	0.03 ^a A^	12.12	±	0.06 ^ab A^	9.40	±	0.03 ^ab A^
	5	12.33	±	0.79 ^ab A^	9.44	±	0.09 ^a A^	11.91	±	0.90 ^ab A^	9.41	±	0.04 ^a A^	12.01	±	0.17 ^ab A^	9.38	±	0.02 ^b A^
	10	14.11	±	1.76 ^a A^	9.45	±	0.04 ^a A^	13.45	±	0.23 ^a A^	9.41	±	0.03 ^a A^	13.39	±	0.92 ^a A^	9.38	±	0.05 ^b A^
*Brochothrix thermosphacta*	0	5.73	±	0.20 ^b^	8.41	±	0.05 ^a^	5.73	±	0.17 ^c^	5.73	±	0.17 ^c^	5.73	±	0.17 ^c^	8.41	±	0.05 ^a^
	1	6.00	±	0.65 ^b A^	8.39	±	0.02 ^a A^	6.46	±	0.41 ^b^.^c A^	6.46	±	0.41 ^b^.^c A^	6.17	±	0.13 ^c A^	8.40	±	0.01 ^a A^
	2	6.60	±	0.62 ^b A^	8.38	±	0.03 ^a A^	6.70	±	0.31 ^b^.^c A^	6.70	±	0.31 ^b^.^c A^	6.41	±	0.07 ^b^.^c A^	8.40	±	0.03 ^a A^
	3	6.74	±	0.51 ^a^.^b A^	8.38	±	0.03 ^a A^	7.40	±	0.49 ^a^.^b A^	7.40	±	0.49 ^a^.^b A^	7.29	±	0.46 ^a A^	8.40	±	0.03 ^a A^
	5	7.08	±	0.28 ^a^.^b A^	8.39	±	0.04 ^a A^	7.71	±	0.35 ^a A^	7.71	±	0.35 ^a A^	7.21	±	0.16 ^a^.^b A^	8.43	±	0.01 ^a A^
	10	8.11	±	0.06 ^a A^	8.39	±	0.04 ^a A^	7.71	±	0.51 ^a A^	7.71	±	0.51 ^a A^	7.62	±	0.28 ^a A^	8.44	±	0.03 ^a A^
*Lactobacillus sakei*	0	18.45	±	1.89 ^b^	8.53	±	0.09 ^a^	18.45	±	1.89 ^c^	8.53	±	0.09 ^a^	18.45	±	1.89 ^d^	8.53	±	0.09 ^a^
	1	22.90	±	1.14 ^a^.^b A^	8.52	±	0.07 ^a A^	24.40	±	1.00 ^a^.^b A^	8.52	±	0.08 ^a A^	22.56	±	0.39 ^c^.^d A^	8.52	±	0.06 ^a A^
	2	22.33	±	1.67 ^a A^	8.53	±	0.02 ^a A^	24.65	±	2.21 ^a^.^b A^	8.53	±	0.05 ^a A^	24.40	±	2.31 ^b^.^c A^	8.52	±	0.07 ^a A^
	3	24.40	±	1.52 ^a A^	8.54	±	0.06 ^a A^	23.77	±	1.38 ^b A^	8.53	±	0.04 ^a A^	26.76	±	2.11 ^b A^	8.53	±	0.05 ^a A^
	5	25.19	±	1.40 ^a A^	8.52	±	0.10 ^a A^	27.18	±	0.93 ^a^.^b A^	8.51	±	0.07 ^a A^	26.94	±	1.02 ^b A^	8.54	±	0.02 ^a A^
	10	25.62	±	1.31 ^a B^	8.52	±	0.05 ^a A^	27.66	±	0.07 ^a B^	8.53	±	0.05 ^a A^	31.31	±	0.90 ^a A^	8.53	±	0.10 ^a A^
*Listeria monocytogenes*	0	10.67	±	0.35 ^a^	8.12	±	0.05 ^a^	10.67	±	0.35 ^a^	8.12	±	0.05 ^a^	10.67	±	0.35 ^a^	8.12	±	0.05 ^a^
	1	10.99	±	0.30 ^a B^	8.12	±	0.04 ^a A^	10.70	±	0.22 ^a B^	8.12	±	0.06 ^a A^	11.76	±	0.02 ^a A^	7.80	±	0.08 ^b^.^c B^
	2	11.05	±	0.21 ^a A^.^B^	8.10	±	0.03 ^a A^	10.70	±	0.48 ^a B^	8.06	±	0.05 ^a A^	11.58	±	0.18 ^a A^	7.88	±	0.06 ^b B^
	3	11.04	±	0.47 ^a A^.^B^	8.04	±	0.07 ^a^.^b A^	10.74	±	0.05 ^a B^	8.10	±	0.06 ^a A^	11.59	±	0.15 ^a A^	7.71	±	0.07 ^c B^
	5	11.08	±	0.08 ^a A^	7.93	±	0.05 ^b A^	10.71	±	1.05 ^a A^	8.02	±	0.08 ^a A^	11.60	±	0.38 ^a A^	7.65	±	0.06 ^c^.^d B^
	10	11.25	±	0.24 ^a A^	7.96	±	0.04 ^b A^	10.70	±	0.17 ^a B^	8.03	±	0.07 ^a A^	11.59	±	0.14 ^b A^	7.55	±	0.06 ^d B^
**Gram-Negative Bacteria**																			
*Moraxella osloensis*	0	7.64	±	0.16 ^b^	8.49	±	0.04 ^a^	7.64	±	0.16 ^c^	8.49	±	0.04 ^a^	7.64	±	0.16 ^b^	8.49	±	0.04 ^a^
	1	7.56	±	0.16 ^b B^	8.51	±	0.06 ^a A^	7.72	±	0.18 ^c B^	8.49	±	0.04 ^a A^	13.77	±	0.98 ^a A^	7.89	±	0.24 ^b A^
	2	7.68	±	0.82 ^b A^	8.50	±	0.18 ^a A^	7.86	±	0.08 ^c A^	8.52	±	0.06 ^a A^	No growth					
	3	7.76	±	0.57 ^b A^	8.48	±	0.10 ^a A^	8.33	±	0.16 ^b^.^c A^	8.51	±	0.04 ^a A^	No growth					
	5	8.05	±	0.30 ^a^.^b B^	8.49	±	0.03 ^a A^	10.04	±	0.81 ^b A^	8.53	±	0.04 ^a A^	No growth					
	10	8.53	±	0.33 ^a B^	8.48	±	0.06 ^a A^	13.74	±	0.94 ^a A^	8.52	±	0.06 ^a A^	No growth					
*Pseudomonas fragi*	0	6.58	±	0.36 ^c^	9.73	±	0.10 ^a^	6.58	±	0.36 ^c^	9.73	±	0.10 ^a^	6.58	±	0.36 ^e^	9.73	±	0.10 ^a^
	1	7.47	±	0.27 ^b B^	9.61	±	0.02 ^b A^	10.29	±	1.21 ^b A^	9.67	±	0.06 ^a A^	6.50	±	0.12 ^e B^	9.37	±	0.04 ^b B^
	2	7.28	±	0.24 ^b C^	9.61	±	0.03 ^b B^	10.27	±	0.43 ^b A^	9.75	±	0.02 ^a A^	9.08	±	1.43 ^d A^.^B^	9.34	±	0.05 ^b^.^c C^
	3	7.33	±	0.22 ^b B^	9.60	±	0.03 ^b B^	13.08	±	1.41 ^a^.^b B^	9.67	±	0.03 ^a A^	18.31	±	0.77 ^c A^	9.25	±	0.05 ^b^.^c C^
	5	9.87	±	1.47 ^a C^	9.58	±	0.04 ^b A^	15.65	±	0.65 ^a B^	9.64	±	0.02 ^a A^	22.42	±	2.65 ^b A^	8.94	±	0.50 ^c B^
	10	10.55	±	1.48 ^a C^	9.61	±	0.02 ^b A^.^B^	16.26	±	2.38 ^a B^	9.71	±	0.09 ^a A^	29.60	±	1.31 ^a A^	7.93	±	0.07 ^d C^
*Acinetobacter baumanii*	0	8.14	±	0.43 ^c^	9.33	±	0.02 ^a^	8.14	±	0.43 ^a^	9.33	±	0.02 ^a^	8.14	±	0.43 ^b^	9.33	±	0.02 ^a^
	1	8.50	±	0.51 ^b^.^c A^	9.34	±	0.02 ^a A^	8.66	±	0.91 ^a A^	9.33	±	0.01 ^a A^	8.64	±	0.50 ^b A^	9.27	±	0.02 ^b B^
	2	8.53	±	0.26 ^b^.^c A^	9.33	±	0.01 ^a A^	8.78	±	0.81 ^a A^	9.33	±	0.02 ^a A^	8.58	±	0.10 ^b A^	9.20	±	0.03 ^c B^
	3	8.39	±	0.23 ^b^.^c A^	9.33	±	0.02 ^a A^	8.88	±	0.20 ^a A^	9.34	±	0.04 ^a A^	8.70	±	0.06 ^b A^	9.19	±	0.01 ^c^.^d B^
	5	9.44	±	0.84 ^a^.^b A^	9.34	±	0.02 ^a A^	9.11	±	0.46 ^a A^	9.34	±	0.02 ^a A^	9.10	±	0.42 ^b A^	9.17	±	0.01 ^d B^
	10	10.24	±	0.39 ^a B^	9.34	±	0.01 ^a A^	8.99	±	0.54 ^a B^	9.35	±	0.03 ^a A^	26.65	±	0.73 ^a A^	9.10	±	0.02 ^e B^
*Escherichia coli*	0	4.28	±	0.27 ^b^	9.00	±	0.01 ^a^	4.28	±	0.27 ^c^	9.00	±	0.01 ^a^	4.28	±	0.27 ^b^	9.00	±	0.01 ^a^
	1	5.33	±	0.56 ^a^.^b A^	8.99	±	0.02 ^a A^	5.69	±	0.05 ^b A^	8.99	±	0.02 ^a^	6.69	±	0.57 ^a A^	8.99	±	0.02 ^a A^
	2	5.00	±	0.12 ^b C^	9.01	±	0.01 ^a A^	5.65	±	0.12 ^b B^	9.00	±	0.01 ^a A^	7.07	±	0.07 ^a A^	9.00	±	0.03 ^a A^
	3	5.31	±	0.63 ^a^.^b B^	9.01	±	0.02 ^a A^	7.07	±	0.51 ^a A^	8.99	±	0.02 ^a A^	6.94	±	0.04 ^a A^	9.00	±	0.02 ^a A^
	5	5.39	±	0.32 ^a^.^b B^	9.01	±	0.02 ^a A^	7.31	±	0.16 ^a A^	9.00	±	0.01 ^a A^	7.13	±	0.02 ^a A^	8.96	±	0.03 ^a^.^b B^
	10	6.14	±	0.46 ^a B^	9.01	±	0.02 ^a A^	7.63	±	0.47 ^a A^	9.01	±	0.02 ^a A^	7.13	±	0.01 ^a A^.^B^	8.94	±	0.02 ^b B^
*Enterobacter aerogenes*	0	5.48	±	0.12 ^c^	9.32	±	0.02 ^a^	5.48	±	0.12 ^c^	9.32	±	0.02 ^a^	5.48	±	0.12 ^b^	9.32	±	0.02 ^a^
	1	5.86	±	0.11 ^b A^	9.35	±	0.03 ^a A^	5.77	±	0.24 ^b^.^c A^	9.34	±	0.03 ^a A^	5.79	±	0.06 ^a^.^b A^	9.33	±	0.02 ^a A^
	2	5.83	±	0.10 ^b A^	9.35	±	0.03 ^a A^	5.84	±	0.35 ^a^.^b^.^c A^	9.33	±	0.02 ^a A^	6.11	±	0.27 ^a A^	9.32	±	0.02 ^a A^
	3	6.02	±	0.07 ^a^.^b A^	9.35	±	0.04 ^a A^	5.82	±	0.20 ^a^.^b^.^c A^	9.33	±	0.02 ^a A^	5.83	±	0.09 ^a A^	9.30	±	0.03 ^a^.^b A^
	5	6.13	±	0.06 ^b A^	9.35	±	0.03 ^a A^	6.00	±	0.02 ^a^.^b A^	9.32	±	0.03 ^a A^	6.11	±	0.18 ^a A^	9.26	±	0.02 ^b B^
	10	6.10	±	0.05 ^a^.^b A^	9.33	±	0.02 ^a A^	6.26	±	0.29 ^a A^	9.31	±	0.04 ^a A^	5.01	±	0.07 ^a A^	9.26	±	0.02 ^b B^
*Salmonella enterica*	0	5.84	±	0.23 ^c^	9.93	±	0.02 ^a^	5.84	±	0.23 ^c^	9.93	±	0.04 ^a^	5.84	±	0.23 ^c^	9.93	±	0.02 ^a^
	1	6.31	±	0.11 ^b A^	9.95	±	0.03 ^a A^	6.41	±	0.28 ^b A^	9.94	±	0.02 ^a A^	6.19	±	0.12 ^b^.^c A^	9.88	±	0.05 ^a^.^b A^
	2	6.55	±	0.13 ^a^.^b A^	9.94	±	0.02 ^a A^	6.41	±	0.17 ^b A^	9.95	±	0.03 ^a A^	6.27	±	0.08 ^b^.^c A^	9.89	±	0.04 ^a^.^b A^
	3	6.58	±	0.22 ^a^.^b A^	9.92	±	0.04 ^a A^	6.64	±	0.07 ^a^.^b A^	9.96	±	0.04 ^a A^	6.52	±	0.13 ^a^.^b A^	9.83	±	0.03 ^c B^
	5	6.90	±	0.02 ^a A^	9.95	±	0.03 ^a A^	6.71	±	0.08 ^a^.^b A^.^B^	9.93	±	0.02 ^a A^	6.51	±	0.28 ^a^.^b B^	9.86	±	0.03 ^b^.^c B^
	10	6.90	±	0.11 ^a A^	9.95	±	0.04 ^a A^	6.82	±	0.06 ^a A^	9.95	±	0.03 ^a A^	6.74	±	0.07 ^a A^	9.80	±	0.04 ^c B^

The results are expressed as mean ± SD, ^a,b,c^–statistically significant differences between different concentrations of the extract (*p* < 0.05). ^A,B,C^–statistically significant differences between the same concentration of different extracts (*p* < 0.05).

**Table 5:** Bacterial strains used in microbiological studies.

Bacterial Strain	Source	Collection Number/Accession Number
**Gram-Positive Bacteria**		
*Enterococcus faecium* WR1	Water	MG911720
*Staphylococcus aureus*	ŁOCK	0891
*Brochothrix thermosphacta* MMAP4	Meat packed in a modified atmosphere	HQ890943.1
*Lactobacillus sakei*	ATCC	15521
*Listeria monocytogenes*	ATCC	13992
**Gram-Negative Bacteria**		
*Moraxella osloensis*	ATCC	10973
*Pseudomonas fragi*	ATCC	4973
*Acinetobacter baumanii*	ATCC	19606
*Escherichia coli*	ATCC	10536
*Enterobacter aerogenes*	PCM	532
*Salmonella enterica* MCH1	Chicken meat	MG911721

## Supplementary Materials

The following are available online, Table S1: Effect of leaf extracts on the maximum growth rate (µ_max_) of bacteria.
