# Supplementary material for: Composition and Antibacterial Activity of Aronia melanocarpa (Michx.) Elliot, Cornus mas L. and Chaenomeles superba Lindl. Leaf Extracts
Source: Molecules. 2020 Apr 25;25(9):2011. doi: 10.3390/molecules25092011 (PMC7248868; doi:10.3390/molecules25092011)
Supplement: Supplementary file 1 [file molecules-25-02011-s001.pdf]

Table S1. Effect of leaf extracts on the maximum growth rate (μ_{max}) of bacteria

Bacteria strain	Extract concentration [%]	*Aronia melanocarpa* μ_{max} [h^{-1}]	*Cornus mas* μ_{max} [h^{-1}]	*Chaenomeles superba* μ_{max} [h^{-1}]
**Gram-positive bacteria**				
Enterococcus faecium	0	0,360 ± 0,032^{a}	0,360 ± 0,032^{a}	0,360 ± 0,032^{a}
	1	0,291 ± 0,027$^{b\ A}$	0,325 ± 0,024$^{a,b\ A}$	0,306 ± 0,055$^{a,b\ A}$
	2	0,280 ± 0,012$^{b\ A}$	0,277 ± 0,039$^{b,c\ A}$	0,308 ± 0,028$^{a,b\ A}$
	3	0,264 ± 0,039$^{b,c\ A}$	0,271 ± 0,015$^{b,c\ A}$	0,234 ± 0,011$^{b,c\ A}$
	5	0,273 ± 0,014$^{b\ A}$	0,242 ± 0,011$^{c\ B}$	0,191 ± 0,016$^{c\ C}$
	10	0,202 ± 0,020$^{c\ A}$	0,172 ± 0,010$^{d\ A}$	0,183 ± 0,008$^{c\ A}$
Staphylococcus aureus	0	0,499 ± 0,066^{a}	0,499 ± 0,066^{a}	0,499 ± 0,066^{a}
	1	0,364 ± 0,032$^{b\ A}$	0,388 ± 0,042$^{ab\ A}$	0,363 ± 0,039$^{b\ A}$
	2	0,361 ± 0,041$^{b\ A}$	0,372 ± 0,003$^{b\ A}$	0,350 ± 0,057$^{b\ A}$
	3	0,300 ± 0,011$^{bc\ A}$	0,335 ± 0,003$^{b\ A}$	0,346 ± 0,039$^{b\ A}$
	5	0,274 ± 0,028$^{bc\ A}$	0,330 ± 0,029$^{b\ A}$	0,325 ± 0,018$^{b\ A}$
	10	0,238 ± 0,029$^{c\ B}$	0,295 ± 0,014$^{b\ A}$	0,327 ± 0,015$^{b\ A}$
Brochothrix thermosphacta	0	0,341 ± 0,004^{a}	0,341 ± 0,004^{a}	0,341 ± 0,004^{a}
	1	0,254 ± 0,024$^{b\ A}$	0,230 ± 0,017$^{b\ A}$	0,242 ± 0,009$^{b\ A}$
	2	0,236 ± 0,007$^{b,c\ A}$	0,219 ± 0,013$^{b\ A}$	0,240 ± 0,006$^{b\ A}$
	3	0,197 ± 0,017$^{b,c\ A}$	0,216 ± 0,007$^{b\ A}$	0,182 ± 0,013$^{c\ A}$
	5	0,193 ± 0,024$^{b,c\ A}$	0,170 ± 0,011$^{c\ A}$	0,181 ± 0,008$^{c\ A}$
	10	0,168 ± 0,033$^{c\ A}$	0,166 ± 0,016$^{c\ A}$	0,138 ± 0,013$^{d\ A}$
Lactobacillus sakei	0	0,232 ± 0,032^{a}	0,232 ± 0,032^{a}	0,232 ± 0,032^{a}
	1	0,220 ± 0,026$^{a\ A}$	0,222 ± 0,013$^{a\ A}$	0,237 ± 0,047$^{a\ A}$
	2	0,220 ± 0,012$^{a\ A}$	0,216 ± 0,016$^{a\ A}$	0,234 ± 0,092$^{a\ A}$
	3	0,223 ± 0,023$^{a\ A}$	0,131 ± 0,019$^{b\ B}$	0,222 ± 0,013$^{a\ A}$
	5	0,227 ± 0,011$^{a\ A}$	0,135 ± 0,020$^{b\ B}$	0,212 ± 0,010$^{a\ A}$
	10	0,194 ± 0,014$^{a\ A}$	0,123 ± 0,046$^{b\ B}$	0,189 ± 0,017$^{a\ A,B}$
Listaeria monocytogenes	0	0,477 ± 0,038^{a}	0,477 ± 0,038^{a}	0,477 ± 0,038^{a}
	1	0,468 ± 0,032$^{a\ A}$	0,438 ± 0,021$^{a,b\ A}$	0,474 ± 0,042$^{a\ A}$
	2	0,452 ± 0,035$^{a\ A}$	0,367 ± 0,037$^{b\ B}$	0,411 ± 0,037$^{a,b\ A,B}$
	3	0,461 ± 0,040$^{a\ A}$	0,245 ± 0,030$^{c\ B}$	0,383 ± 0,042$^{b,c\ A}$
	5	0,465 ± 0,005$^{a\ A}$	0,170 ± 0,032$^{c,d\ C}$	0,306 ± 0,008$^{c,d\ B}$
	10	0,447 ± 0,057$^{a\ A}$	0,129 ± 0,035$^{d\ C}$	0,249 ± 0,008$^{d\ B}$
**Gram-negative bacteria**				
Moraxella osloensis	0	0,150 ± 0,013^{a}	0,150 ± 0,013^{a}	0,150 ± 0,013^{a}
	1	0,134 ± 0,004$^{a,b\ A}$	0,019 ± 0,006$^{b\ C}$	0,094 ± 0,008$^{b\ B}$
	2	0,114 ± 0,001$^{b,c\ A}$	No growth	0,100 ± 0,007$^{b\ A}$
	3	0,114 ± 0,015$^{b,c\ A}$	No growth	0,057 ± 0,010$^{c\ B}$
	5	0,105 ± 0,015$^{c,d\ A}$	No growth	0,050 ± 0,006$^{c\ B}$
	10	0,084 ± 0,011$^{d\ A}$	No growth	0,055 ± 0,005$^{c\ B}$
Pseudomonas fragi	0	0,120 ± 0,006^{a}	0,120 ± 0,006^{a}	0,120 ± 0,006$^{a\ A}$
	1	0,116 ± 0,005$^{a\ A}$	0,111 ± 0,010$^{a\ A}$	0,102 ± 0,016$^{a\ A}$
	2	0,115 ± 0,006$^{a\ A}$	0,060 ± 0,008$^{b\ C}$	0,095 ± 0,006$^{a\ B}$
	3	0,112 ± 0,006$^{a\ A}$	0,026 ± 0,003$^{c\ B}$	0,109 ± 0,013$^{a\ A}$
	5	0,095 ± 0,005$^{b\ B}$	0,025 ± 0,004$^{c\ C}$	0,115 ± 0,012$^{a\ A}$
	10	0,095 ± 0,011$^{b\ A}$	0,025 ± 0,002$^{c\ B}$	0,104 ± 0,017$^{a\ A}$
Acinetobacter baumanii	0	0,206 ± 0,015^{a}	0,206 ± 0,015^{a}	0,206 ± 0,015^{a}
	1	0,185 ± 0,001$^{a,b,c\ A}$	0,120 ± 0,021$^{b\ B}$	0,180 ± 0,003$^{a\ A}$
	2	0,189 ± 0,004$^{a,b\ A}$	0,105 ± 0,013$^{b,c\ B}$	0,178 ± 0,016$^{a\ A}$
	3	0,171 ± 0,009$^{b,c\ A}$	0,093 ± 0,014$^{b,c\ B}$	0,178 ± 0,013$^{a\ A}$
	5	0,165 ± 0,008$^{b,c\ A}$	0,082 ± 0,003$^{c\ B}$	0,173 ± 0,007$^{a,b\ A}$
	10	0,161 ± 0,011$^{c\ A}$	0,095 ± 0,014$^{b,c\ B}$	0,140 ± 0,018$^{b\ A}$

Escherichia coli	0	0,556 ± 0,050[a]	0,556 ± 0,050[a]	0,556 ± 0,050[a]
	1	0,398 ± 0,010[b A,B]	0,483 ± 0,024[b A]	0,375 ± 0,029[b B]
	2	0,310 ± 0,025[c B]	0,160 ± 0,022[c C]	0,365 ± 0,023[b A]
	3	0,285 ± 0,019[c B]	0,124 ± 0,005[c,d C]	0,351 ± 0,053[b A]
	5	0,273 ± 0,009[c B]	0,099 ± 0,007[d,e C]	0,301 ± 0,018[b A]
	10	0,267 ± 0,009[c A]	0,065 ± 0,004[e B]	0,288 ± 0,014[b A]
Enterobacter aerogenes	0	0,369 ± 0,039[a]	0,369 ± 0,039[a]	0,369 ± 0,039[a]
	1	0,369 ± 0,015[a A]	0,285 ± 0,036[b B]	0,275 ± 0,005[b B]
	2	0,325 ± 0,020[a,b A]	0,162 ± 0,010[c C]	0,273 ± 0,004[b B]
	3	0,323 ± 0,003[a,b A]	0,144 ± 0,004[c C]	0,256 ± 0,032[b B]
	5	0,295 ± 0,014[b A]	0,118 ± 0,010[c C]	0,257 ± 0,003[b B]
	10	0,275 ± 0,002[b A]	0,060 ± 0,010[d C]	0,243 ± 0,003[b B]
Salmonella enterica	0	0,227 ± 0,005[a]	0,227 ± 0,005[a]	0,227 ± 0,005[a]
	1	0,176 ± 0,017[b A]	0,180 ± 0,005[b A]	0,169 ± 0,014[b A]
	2	0,147 ± 0,014[b,c B]	0,181 ± 0,005[b A]	0,165 ± 0,003[b,c A,B]
	3	0,141 ± 0,015[c,d B]	0,153 ± 0,007[c A,B]	0,165 ± 0,004[b,c A]
	5	0,116 ± 0,007[d,e B]	0,153 ± 0,004[c A]	0,161 ± 0,004[b,c A]
	10	0,104 ± 0,006[e C]	0,124 ± 0,004[d B]	0,150 ± 0,004[c A]

The results are expressed as mean ± SD

[a,b,c] – statisticaly significant differences between different concentrations of the extract ($p<0.05$)

[A, B, C] – statistically significant differences between the same concentration of different extracts ($p<0.05$)
